# CORRIGENDUM

**DOI:** 10.1002/mpr.1907

**Published:** 2022-06-27

1

Dear Editor,

We would like to submit this corrigendum regarding our previously published manuscript (Mapping of the World Health Organization's Disability Assessment Schedule 2.0 to disability weights using the Multi‐Country Survey Study on Health and Responsiveness; https://pubmed.ncbi.nlm.nih.gov/34245195/), as we found some errors in the parameter values that make up the mapping function, constituting the main finding of our article. This results in the following changes in the article:

– A replacement of the values in the Tables 2, 3, 4–2, 3, 4;
– A replacement of Figure 2;
– A replacement of the numbers in the text;

**TABLE 2:** Model performance of the various statistical learning models predicting disability weights

Model nr.	WHODAS version	Method	Predictors included in model/algorithm	RMSE	*R* ^2^	RMSE (Test set)^b^	*R* ^2^ (Test set)^c^
1	36‐item	Linear regression	Individual items	0.050	0.694		
2	**36‐item**	**Linear regression**	**Individual items & demographics** ^a^	**0.050**	**0.692**	**0.052**	**0.683**
3	36‐item	Linear regression	All six domain scores	0.056	0.613		
4	36‐item	Linear regression	All six domain scores & demographics^a^	0.056	0.610		
5	36‐item	Linear regression	Individual items, demographics^a^ & country dummy	0.05	0.697		
6	**36‐item**	**Linear regression**	**Individual items, demographics** ^a^ **, country dummy, all country interactions**	**0.053**	**0.675**	**0.049**	**0.719**
7	12‐item	Linear regression	Individual items	0.054	0.645		
8	**12‐item**	**Linear regression**	**Individual items & demographics** ^a^	**0.054**	**0.646**	**0.056**	**0.636**
9	12‐item	Linear regression	Individual items, demographics^a^ & country dummy	0.054	0.651		
10	**12‐item**	**Linear regression**	**Individual items, demographics** ^a^ **, country dummy, all country interactions**	**0.057**	**0.624**	**0.052**	**0.681**
11	36‐item	LASSO regression	Individual items	0.050	0.693		
12	**36‐item**	**LASSO regression**	**Individual items & demographics** ^a^	**0.050**	**0.692**	**0.052**	**0.682**
13	36‐item	LASSO regression	All six domain scores	0.056	0.613		
14	36‐item	LASSO regression	All six domain scores & demographics^a^	0.056	0.612		
15	36‐item	LASSO regression	Individual items, demographics^a^ & country dummy	0.050	0.696		
16	**36‐item**	**LASSO regression**	**Individual items, demographics** ^a^ **, country dummy, all country interactions**	**0.051**	**0.696**	**0.050**	**0.708**
17	12‐item	LASSO regression	Individual items	0.054	0.645		
18	**12‐item**	**LASSO regression**	**Individual items & demographics** ^a^	**0.054**	**0.646**	**0.056**	**0.636**
19	12‐item	LASSO regression	Individual items, demographics^a^ & country dummy	0.054	0.651		
20	**12‐item**	**LASSO regression**	**Individual items, demographics** ^a^ **, country dummy, all country interactions**	**0.055**	**0.648**	**0.054**	**0.659**

*Note*: Per WHODAS version, per statistical learning method, and for the models with and without country information, the best performing model is bold faced.

^a^ Demographic variables include: age, gender, educational level, and marital status

^b^ Root‐mean‐squared error for each model predicting disability weights using WHODAS responses on the test set

^c^ *R*‐squared for each model predicting disability weights using WHODAS responses on the test set

**TABLE 3:** Mapping function for WHODAS 2.0‐36 and WHODAS 2.0‐12 with demographics based on LASSO regression

Predictor	Model 12 (WHODAS 2.0‐36)^a^ ^,^ ^b^	Model 18 (WHODAS 2.0‐12)^a^ ^,^ ^b^
Intercept	0.1055	0.1021
Items in original WHODAS 2.0‐36 / WHODAS 2.0‐12		
D1.1/S6	0.0428	0.0464
D1.4/S3	‐	0.0009
D1.5	‐	NA
D1.6	−0.0002	NA
D2.2	0.0162	NA
D2.3	0.0179	NA
D3.1/S8	0.0220	0.0317
D3.2/S9	0.0089	0.0183
D3.4	0.0019	NA
D4.2/S11	‐	‐
D4.3	0.0002	NA
D4.5	‐	NA
D5.1/S2	0.0052	0.0179
D5.3	0.0107	NA
D5.5/S12	0.0042	0.0137
D5.7	‐	NA
D6.1/S4	0.0047	0.0098
D6.6	0.0042	NA
D6.7	0.0096	NA
Demographic variables		
Age	0.0000	0.0003
Gender (male)	−0.0026	−0.0052
Education (high school or equivalent)	0.0015	‐
Marital status (widowed)	0.0020	0.0044

^a^ For model specifications see Table 2

^b^ All WHODAS items are converted to a 0‐4 scale

**TABLE 4:** Comparison model performance of generic model to country‐specific models on country‐specific test sets

	Generic model – *R*‐squared	Country‐specific model – *R*‐squared	’Other‐countries’ model – *R*‐squared^b^
Model 12 (WHODAS 2.0‐36)^a^			
China (*N* = 9486)	0.677	0.682	0.655
Colombia (*N* = 8158)	0.623	0.605	0.613
Egypt (*N* = 4490)	0.758	0.749	0.745
Georgia (*N* = 9847)	0.793	0.766	0.782
Indonesia (*N* = 9994)	0.653	0.522	0.549
India (*N* = 5144)	0.758	0.753	0.752
Iran (*N* = 9718)	0.697	0.687	0.684
Lebanon (*N* = 3246)	0.767	0.713	0.748
Mexico (*N* = 4813)	0.697	0.595	0.683
Nigeria (*N* = 5108)	0.537	0.521	0.637
Singapore (*N* = 6216)	0.676	0.642	0.790
Slovakia (*N* = 1183)	0.780	0.757	0.782
Syria (*N* = 9344)	0.684	0.660	0.665
Turkey (*N* = 5207)	0.652	0.611	0.624
Model 18 (WHODAS 2.0‐12)^a^			
China (*N* = 9486)	0.648	0.636	0.579
Colombia (*N* = 8158)	0.586	0.561	0.575
Egypt (*N* = 4490)	0.729	0.708	0.719
Georgia (*N* = 9847)	0.763	0.736	0.738
Indonesia (*N* = 9994)	0.619	0.465	0.493
India (*N* = 5144)	0.699	0.682	0.687
Iran (*N* = 9718)	0.646	0.655	0.651
Lebanon (*N* = 3246)	0.735	0.666	0.715
Mexico (*N* = 4813)	0.661	0.585	0.659
Nigeria (*N* = 5108)	0.480	0.468	0.594
Singapore (*N* = 6216)	0.629	0.522	0.690
Slovakia (*N* = 1183)	0.739	0.716	0.741
Syria (*N* = 9344)	0.628	0.579	0.602
Turkey (*N* = 5207)	0.606	0.571	0.571

^a^ For model specifications see Table 2

^b^ Performance of models trained on data from the 13 other countries

The details of these changes are outlined below. As discussed, this corrigendum should replace the one which was pending.

Thank you very much for your consideration, and please let us know if additional input is required.

On behalf of the co‐authors,

Dr. Joran Lokkerbol

New Table 2

New Table 3

New Table 4

New Figure 2

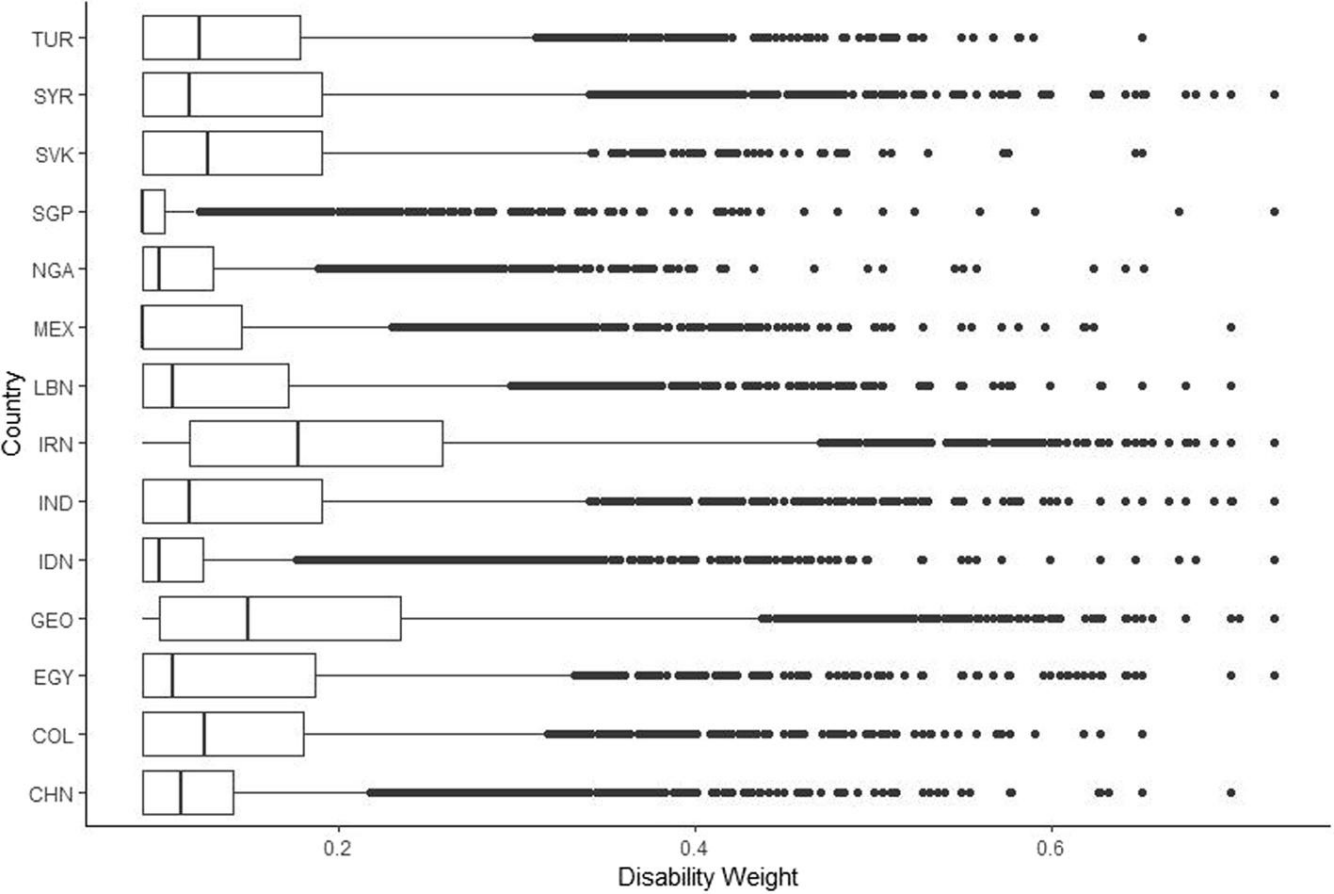

**Changes in numbers in the text (changes highlighted in bold)**

***Changes in the Abstract (‘results’ header)***

***Old text*:**

Results: Mapping functions converted WHODAS‐2.0 scores into disability weights; R‐squared values of 0.700–0.754 were obtained for the test data set. Penalized regression models reached comparable performance to standard regression models but with fewer predictors. Imputation had little impact on model performance. Model performance of the generic model on country‐specific test sets was com‐ parable to model performance of country‐specific models.

***New text*:**

Results: Mapping functions converted WHODAS‐2.0 scores into disability weights; R‐squared values of **0.636–0.719** were obtained for the test data set. Penalized regression models reached comparable performance to standard regression models but with fewer predictors. Imputation had little impact on model performance. Model performance of the generic model on country‐specific test sets was com‐ parable to model performance of country‐specific models.

**Changes in the Results (‘3.2 disability weight’ header)**

***Old text*:**

Mean disability weight was 0.12 (SD: 0.05), ranging from 0.17 (SD: 0.10) in the Iran dataset to 0.10 (SD: 0.04) in the Singapore dataset. For all countries, data were right‐skewed with medians ranging from 0.13 (Iran) to 0.09 (Mexico, Nigeria, and Singapore). Distributions of disability weight per country are shown in Figure 2.

***New text*:**

Mean disability weight was **0.15 (SD: 0.09)**, ranging from **0.20 (SD: 0.11)** in the Iran dataset to **0.11 (SD: 0.04)** in the Singapore dataset. For all countries, data were right‐skewed with medians ranging from **0.18** (Iran) to 0.09 (Mexico and Singapore). Distributions of disability weight per country are shown in Figure 2.

**Changes in the Results (‘3.3 Fitting of statistical learning models’ header)**

***Old text*:**

Furthermore, the performance of the linear regression and the LASSO regression was similar (RMSE: 0.040–0.046 and R (Donahue et al., 2018): 0.700–0.754 on the test set).

***New text*:**

Furthermore, the performance of the linear regression and the LASSO regression was similar (RMSE: **0.049–0.056** and ***R* ^2^: 0.636–0.719** on the test set).

**Changes in the Results (‘3.4 Sensitivity analyses’ header)**

***Old text*:**

Model performance for this subsample was lower than in the main analyses, with an *R* ^2^ of 0.645 using WHODAS 2.0–36 and 0.607 using WHODAS 2.0–12.

***New text*:**

Model performance for this subsample was lower than in the main analyses, with an *R* ^2^ of **0.621** using WHODAS 2.0‐36 and **0.582** using WHODAS 2.0‐12.

***Old text*:**

Imputing missing WHODAS domain scores using the mean of those domain scores for other respondents instead of the mean of other domain scores within the same respondent resulted in similar results compared to the main analyses with the alternative analyses leading to slightly lower performance metrices with an *R* ^2^ of 0.738 compared to 0.743 (WHODAS 2.0–36) and 0.692 compared to 0.705 (WHODAS 2.0–12) and comparable RMSEs compared to the base case analyses. Likewise, imputing missing WHODAS domain scores using kNN imputation resulted in similar results compared to the main analyses with an *R* ^2^ of 0.742 compared to 0.743 (WHODAS 2.0–36) and 0.701 compared to 0.705 (WHODAS 2.0–12) and comparable RMSE values. All coefficients had similar signs as in the main analyses. For the country‐specific models, see Table 4, performance varied from an *R* ^2^ of 0.593 (WHODAS 2.0–36; Nigeria) and 0.523 (WHODAS 2.0–12; Nigeria) to 0.811 (WHODAS 2.0–36; Georgia) 0.794 (WHODAS 2.0–12; Georgia).

***New text*:**

Imputing missing WHODAS domain scores using the mean of those domain scores for other respondents instead of the mean of other domain scores within the same respondent resulted in similar results compared to the main analyses with the alternative analyses leading to slightly lower performance metrices with an *R* ^2^ of **0.690** compared to **0.692** (WHODAS 2.0‐36) and **0.641** compared to **0.646** (WHODAS 2.0‐12) and comparable RMSEs compared to the base case analyses. Likewise, imputing missing WHODAS domain scores using kNN imputation resulted in similar results compared to the main analyses with an *R* ^2^ of **0.690** compared to **0.692** (WHODAS 2.0–36) and **0.646, which is similar to the base case analysis** (WHODAS 2.0–12) and comparable RMSE values. All coefficients had similar signs as in the main analyses. For the country‐specific models, see table 4, performance varied from an *R* ^2^ of **0.537** (WHODAS 2.0‐36; Nigeria) and **0.480** (WHODAS 2.0‐12; Nigeria) to **0.766** (WHODAS 2.0‐36; Georgia) **0.736** (WHODAS 2.0‐12; Georgia).

***Old text*:**

The mapping functions presented in Table 3 are straightforward to use for converting WHODAS 2.0 scores into disability weight estimates. As a hypothetical example, assume there is a trial in which patients treated for moderate depression are randomized to either care as usual or care as usual plus additional treatment. In both arms, patients are aged 40 on average at baseline and 50% of patients is female. WHODAS 2.0–36 is administered and patients in both arms score a two on every item of the WHODAS 2.0 (scaled to 0–4 in concordance with the WHODAS scoring manual). This means that at baseline, average disability weight in both groups is 0.38725 (see equation 1). Assume the items D1.1 (Concentrating on doing something for ten mi‐ nutes), D4.2 (Maintaining a friendship), D5.1 (Taking care of your house‐ hold responsibilities), D5.3 (Getting all the household work done that you needed to do), D5.5 (Your day‐to‐day work/school), D5.7 (Getting all the work done that you need to do) and D6.1 (How much of a problem did you have in joining in community activities (for example, festivities, religious or other activities) in the same way as anyone else can) are positively impacted by the treatment, improving from 2 to 1.5 in the care as usual group and from 2 to one in the care as usual plus additional treatment group one year after baseline. This would improve disability weights by an estimated 0.38725–0.2942 = 0.09305 in the additional treatment group and by an estimated 0.38725–0.3400 = 0.0456 in the care as usual group. These improvements in disability weight could then be entered into calculation of QALYs gained.

Disability weight = 0.1344 + 0.0273 ∗ 2 + 0.0011 ∗ 2 + 0.0087 ∗ 2 + 0.0121∗2 + 0.0119∗2 + 0.0042∗2 + 0.0006∗2 + 0.0020∗2 + 0.0001∗2 + 0.0006∗2 + 0.0042∗2 + 0.0072∗2 + 0.0029∗2 + 0.0067∗2 + 0.0010∗2 + 0.0039∗2 + 0.0016∗40 – 0.0003∗0.50

***New text*:**

The mapping functions presented in table 3 are straightforward to use for converting WHODAS 2.0 scores into disability weight estimates. As a hypothetical example, assume there is a trial in which patients treated for moderate depression are randomized to either care as usual or care as usual plus additional treatment. In both arms, patients are aged 40 on average at baseline, **50% of patients is female, everyone finished secondary school and everyone is married**. WHODAS 2.0‐36 is administered and patients in both arms score a 2 on every item of the WHODAS 2.0 (scaled to 0‐4 in concordance with the WHODAS scoring manual). This means that at baseline, average disability weight in both groups is **0.4016** (see equation 1). Assume the items D1.1 (Concentrating on doing something for ten minutes), D4.2 (Maintaining a friendship), D5.1 (Taking care of your household responsibilities), D5.3 (Getting all the household work done that you needed to do), D5.5 (Your day‐to‐day work/school), D5.7 (Getting all the work done that you need to do) and D6.1 (How much of a problem did you have in joining in community activities (for example, festivities, religious or other activities) in the same way as anyone else can) are positively impacted by the treatment, improving from 2 to 1.5 in the care as usual group and from 2 to one in the care as usual plus additional treatment group one year after baseline. This would improve disability weights by an estimated **0.4016** – **0.3340** = **0.0676** in the additional treatment group and by an estimated **0.4016–0.3678** = **0.0338** in the care as usual group. These improvements in disability weight could then be entered into calculation of QALYs gained.

Disability weight = 0.1055 + 0.0428*2 – 0.0002*2 + 0.0162*2 + 0.0179*2 + 0.0220*2 + 0.0089*2 + 0.0019*2 + 0.0002*2 + 0.0052*2 + 0.0107*2 + 0.0042*2 + 0.0047*2 + 0.0042*2 + 0.0096*2 + 0.00002*40 – 0.0026*0.50

**Changes in the Conclusion and Discussion (‘3.3 Fitting of statistical learning models’ header)**

***Old text*:**

By exploring various model specifications, using both linear regression and LASSO regression, we found good model performances (with *R* ^2^ > 0.70 on the test set, including for models not using country‐specific information.

***New text*:**

By exploring various model specifications, using both linear regression and LASSO regression, we found good model performances (with *R* ^2^ > **0.636** on the test set, including for models not using country‐specific information).

***Old text*:**

Sixth, predictions on an individual level can still exhibit quite some variance (the mean disability was 0.13 with a SD of 0.08).

***New text*:**

Sixth, predictions on an individual level can still exhibit quite some variance (the mean disability was **0.15** with a SD of **0.09**).

